# Advancing Healthcare Accessibility: Fusing Artificial Intelligence with Flexible Sensing to Forge Digital Health Innovations

**DOI:** 10.34133/bmef.0062

**Published:** 2024-08-27

**Authors:** Lingting Huang, Zhengjie Chen, Zhen Yang, Wei Huang

**Affiliations:** ^1^ Strait Institute of Flexible Electronics (SIFE, Future Technologies), Fujian Key Laboratory of Flexible Electronics, Fujian Normal University and Strait Laboratory of Flexible Electronics (SLoFE), Fuzhou 350117, China.; ^2^ Frontiers Science Center for Flexible Electronics (FSCFE), MIIT Key Laboratory of Flexible Electronics (KLoFE), Northwestern Polytechnical University, Xi’an 710072, China.

In recent years, the rapid advancement of digital technologies has precipitated a paradigm shift in global healthcare, heralding a new era of digital health methodologies. This transition underscores a universal consensus on the imperative of digitalization and the application of sophisticated information and communication technologies to achieve universal health coverage, aimed at enhancing health outcomes and overall well-being. Central to this transformation is the integration of advanced technologies such as artificial intelligence (AI), big data analytics, wearable smart devices, and the Internet of Things (IoT), which have greatly enhanced data collection, analysis, storage, and transmission, laying the foundation for a comprehensive healthcare system [1,2]. This system significantly improves diagnostic accuracy, enables data-driven therapeutic interventions, supports individual health self-management, and promotes patient-centric care, thus playing a crucial role in the ongoing development of healthcare infrastructures. As digital health continues to evolve, it prompts a focused examination toward eliminating healthcare disparities, aiming for a universal healthcare model that emphasizes accessibility, affordability, and sustainability [3]. Flexible sensing technologies stand out in today’s healthcare system because of their adaptability, comfort, and lightweight construction, which improve data accuracy and continuity for enhanced health monitoring. Their integration with AI represents a significant advancement in personal health management and supports a more inclusive digital health ecosystem, vastly enhancing health data analysis and advancing healthcare equity and accessibility (Figure).

**Figure.**
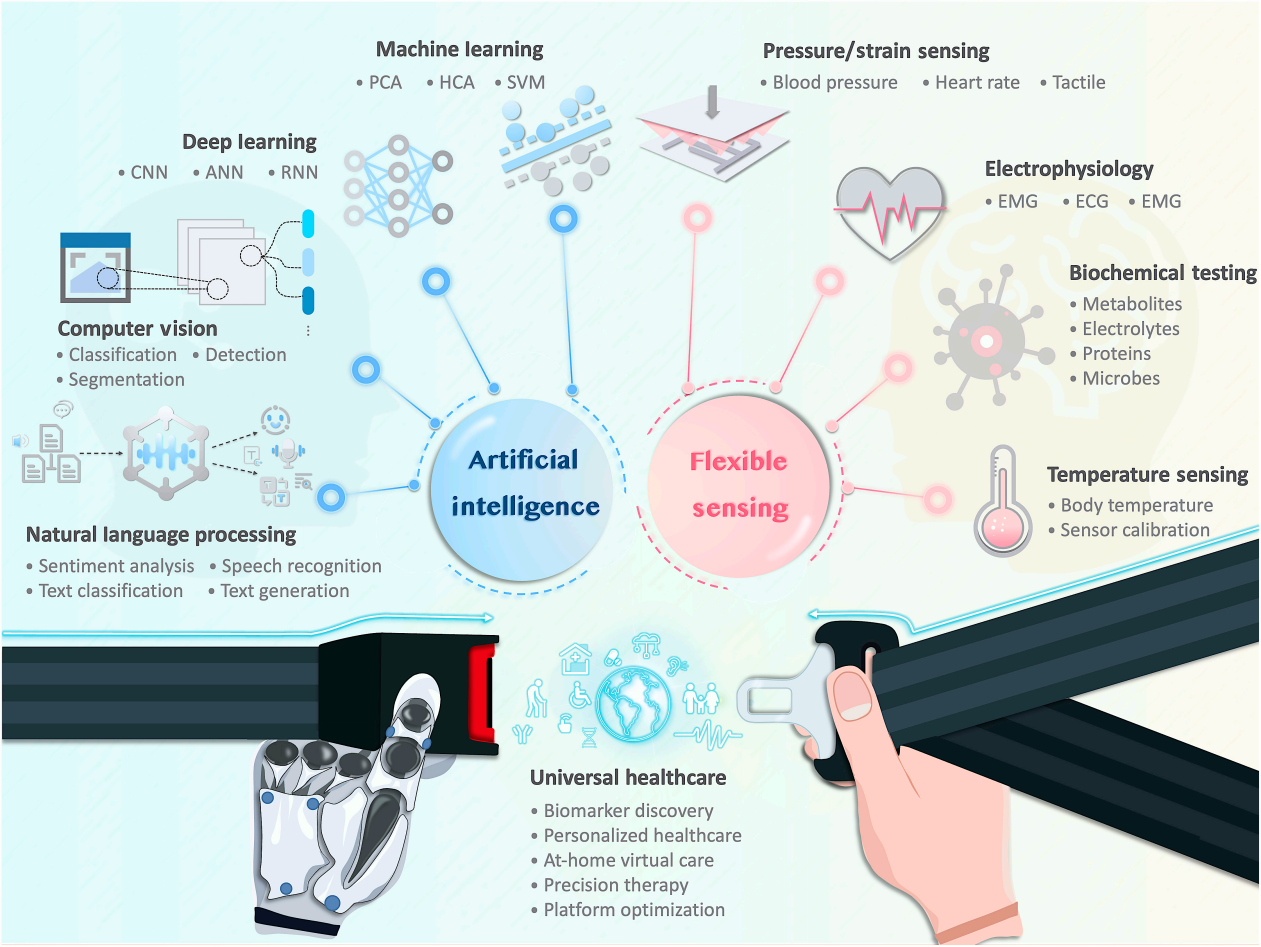
Overview of artificial intelligence and flexible sensing systems.

Constructed from elastic materials designed to conform to the human body’s various shapes, flexible sensors are increasingly utilized in healthcare for monitoring health indicators. These sensors are adept at measuring a comprehensive array of physiological and biochemical markers, such as heart rate, blood pressure, respiratory functions, electrophysiological signals, metabolites, proteins, electrolytes, and microbial presence, showcasing their utility through high compliance, seamless integration, noninvasiveness, and cost-effectiveness [4]. Their application spans remote disease monitoring, therapeutic interventions, intelligent prosthetics, and rehabilitation technologies, thereby opening new avenues in personalized medicine, disease prevention, and health management, enhancing patient quality of life, and reducing healthcare costs [4,5]. Despite the strides made in flexible sensing systems, their development faces significant challenges, including material selection, stability of the human–machine interface, durability over long-term use, performance enhancement, and biocompatibility. Researchers are tackling these obstacles with innovations in material science, refined interface design, and advancements in encapsulation technologies, although this endeavor is resource-intensive and time-consuming. Additionally, as sensors become more miniaturized and integrated, overcoming data inaccuracies induced by bodily movements to ensure data reliability becomes crucial. Furthermore, the management of increasingly complex data, alongside ensuring data security and privacy, poses considerable challenges. Facing a spectrum of challenges from technological development to data protection, system integration, production efficiency, and interdisciplinary collaboration, the advancement of flexible sensors relies on cross-disciplinary innovation aimed at improving technical performance, safeguarding user privacy, and fostering sustainable development. Nevertheless, the ongoing development and increasing cost-effectiveness of these technologies warrant optimism for flexible sensing system as a powerful catalyst in improving global health standards. Particularly valuable in resource-limited regions, the convenience offered by flexible sensors allows users to access real-time health data without requiring specialized knowledge. This significantly facilitates the expansion and improvement of health services worldwide.

With the ongoing advancements in flexible sensing technology, the field of AI has simultaneously experienced rapid development. AI’s capabilities in learning, reasoning, planning, and perception have markedly unveiled its potential to mimic and amplify human cognitive functions, thereby altering our perspective on what computational technologies can achieve. Within the realms of material science and sensor design, AI is catalyzing a revolution via machine learning (ML) techniques, heralding a fundamental shift in materials discovery and design methodologies. For instance, it can facilitate the improvement of conductivity and mechanical flexibility by accelerating screening processes and optimizing performance predictions. This encompasses the procedures of data-driven models to correlate material structures with their performance, guiding the optimization of experimental synthesis and processes, and enhancing the accuracy of databases and models through experimental validation, thereby achieving high precision and repeatability in sensor performance [6]. Compared to traditional electromechanical simulations, AI exhibits higher data processing efficiency and the capacity to interpret complex nonlinear interactions. This makes it possible to extrapolate characteristics of large-scale systems from computational outcomes obtained from small samples [7,8]. Furthermore, the application of AI is anticipated to facilitate the scalable and customized production of flexible sensors [9,10]. Researchers explored combining ML algorithms with finite element analysis to develop devices capable of transitioning from 2-dimensional (2D) planes to pre-programmed 3D constructions. This illustrates the potential of AI in developing ergonomically designed 3D flexible devices. Moreover, AI techniques are transformative in discovering novel biomarkers and offering predictive capabilities for various diseases that currently lack reliable indicators [11,12]. A research team has designed an AI-based system capable of diagnosing and assessing the severity of Parkinson’s disease (PD) by analyzing nocturnal respiratory signals. The results demonstrated a high correlation with conventional clinical assessment methods. This represents a ground-breaking, noninvasive approach to managing PD, providing patients with a less intrusive, yet efficacious monitoring option [12].

In the meantime, AI has profoundly changed the way we manage huge and complicated datasets, especially in multifunctional flexible sensors, where they provide solutions that traditional methods cannot. ML algorithms, which are based on various learning paradigms such as supervised, unsupervised, semi-supervised, and reinforcement learning, can process a variety of data types, including continuous, categorical, and time-series data, and are suitable for a wide range of applications such as prediction, classification, clustering, and anomaly detection. For instance, decision trees stand out in classification and regression for their simplicity and effectiveness, while support vector machines excel in classification, particularly with high-dimensional data and intricate boundary challenges. Deep learning methods, like convolutional neural networks, are particularly adept at processing image and video data, facilitating autonomous feature learning for complex pattern recognition, albeit at the cost of substantial data and computational demands and susceptibility to overfitting. Selecting the appropriate algorithm involves a comprehensive evaluation of data attributes, task requirements, model complexity, and available computational resources. Generally, linear regression, logistic regression, or decision trees are optimal for structured data, whereas deep learning is preferable for unstructured data [13]. For large datasets, algorithms such as random forests or gradient boosting machines are effective. Furthermore, algorithm selection must take into account model interpretability, computational efficiency, and scalability to enable optimal performance and clarity of understanding in specific applications. By employing these advanced ML algorithms, flexible sensors can more precisely interpret physiological and behavioral data, significantly enhancing performance, accelerating data processing speeds, and improving accuracy. In personalized medicine, AI becomes crucial in calibrating nonlinear sensors, reducing detection variances, and significantly enhancing disease prediction and early diagnostic accuracy, making it an essential component of modern medical solutions.

AI is driving the transformation toward a more inclusive healthcare system that delivers personalized care to diverse populations. Powered by AI, flexible sensors have catalyzed a range of innovative applications that have notably enhanced communication and safety. These applications include voice-to-text conversion and sign language recognition, which aid individuals with voice disorders or hearing impairments, as well as environmental sound alerts that contribute to their safety [14,15]. Additionally, these advanced sensors have made reading more accessible to the visually impaired by using speech recognition or tactile feedback systems for Braille [16,17]. They also have the potential to provide prosthetic users with a simulated sense of touch, vastly improving the functionality and user experience of their prosthetic limbs [18]. These advances overcome the linguistic and sensory limitations of traditional medical services, significantly enhancing quality of life and broadening the scope of medical services. Furthermore, AI enhances access to medical care for those who are unable to get to medical institutions by allowing remote medical services that offer remote consultations, diagnostics, and therapies, using AI to evaluate patient data and deliver individualized medical recommendations and treatment plans [19,20]. AI’s ability to process complex multidimensional datasets and identify potential correlations is accelerating the development of precision medicine and personalized treatment techniques, ushering in a new age in healthcare where treatment programs are tailored to individual needs. This achievement not only promotes the accessibility and effectiveness of care for specific populations but also provides new insights into the continually changing healthcare sector, ensuring its adaptability, reactivity, and long-term care.

To sum up, the integration of AI with flexible sensing technologies has significantly propelled the healthcare sector toward greater inclusivity. The crux of this advancement lies in employing cutting-edge learning algorithms and big data analytics, enabling healthcare professionals to devise personalized treatment plans based on individuals’ genetic information, lifestyle habits, and environmental factors. Such customization not only enhances the precision of treatment and prevention but also lays the groundwork for a more efficient healthcare system. The incorporation of flexible sensors further facilitates continuous monitoring of personal health conditions, which is crucial for the early identification and management of health issues, and can seamlessly integrate into daily life, minimizing disruptions.

Although this transformative journey brings significant advancements, it also presents challenges that must be addressed, such as ensuring data privacy and security, mitigating the risk of health-related discrimination, and keeping pace with the rapid technological advancements within the ethical and legal frameworks. Addressing these challenges necessitates a holistic approach involving collaborative global policies, continual technological refinement, interdisciplinary research efforts, and a commitment to fostering ethical awareness. The goal is to ensure that the benefits brought forth by AI and flexible sensors in healthcare are realized in an innovative, equitable manner that aligns with societal values. Ultimately, our vision is to achieve a personalized and efficient healthcare future where high-quality medical services are accessible to all, necessitating a delicate balance among innovation, policy formulation, and ethical considerations.
